# Exploring the feasibility of wavelength modulated near-infrared spectroscopy

**DOI:** 10.1117/1.JBO.25.11.110501

**Published:** 2020-11-04

**Authors:** Jeremy C. Hebden

**Affiliations:** University College London, Department of Medical Physics and Biomedical Engineering, London, United Kingdom

**Keywords:** near-infrared spectroscopy, wavelength modulation, tissue optics, modified Beer–Lambert law

## Abstract

**Significance**: The application of near-infrared spectroscopy (NIRS) to determine the concentrations of tissue chromophores has typically relied on three alternative technological approaches: continuous-wave, frequency-domain, and time-domain. It is often the case that uncertain and variable coupling of light into and out of the skin surface renders absolute measurements unreliable, and NIRS methods are mostly used to measure changes of chromophore concentrations and of physiological parameters such as blood volume and oxygenation.

**Aim**: The aim has been to investigate whether an approach based on a wavelength-modulated source may enable measurements to be acquired, which are independent of surface coupling and may facilitate derivation of absolute values of tissue parameters.

**Approach**: An analysis is performed using the modified Beer–Lambert law.

**Results**: It is shown that the relative modulation in detected intensity resulting from a wavelength-modulated source could be used to estimate absolute concentrations of chromophores if unknown surface coupling losses and geometrical factors are insensitive to small changes in wavelength.

**Conclusions**: Wavelength modulated NIRS could be an effective tool for quantitative *in vivo* analysis of tissues, although it may be technically challenging.

Medical applications of near-infrared spectroscopy (NIRS) involve illuminating the human body with a harmless beam of near-infrared (NIR) light and then measuring the light that emerges at other discrete locations on the surface. Radiation at NIR wavelengths is absorbed differently by oxy- and deoxy-hemoglobin, and thus measurements are sensitive to the concentration and oxygenation of blood within the tissue. For example, so-called functional NIRS has been widely used as a means of observing hemodynamic responses within the cerebral cortex to sensory stimuli or cognitive tasks.^1^ Unfortunately, NIRS is also highly sensitive to the uncertain and variable coupling of light into and out of the skin surface. Although techniques have been explored to calibrate for such losses using phantoms,^2^ NIRS is most often used to measure changes in tissue optical properties and of physiological parameters such as blood volume and oxygenation.

Measurement of very small changes in intensity is much enhanced by modulating the source intensity at a fixed frequency (typically a few kHz) and using a lock-in amplifier (or Fourier analysis) to isolate the signal amplitude at the source frequency. This exploits the fact that noise is often spread over a much broader range of frequencies than the signal, leading to many orders of magnitude of improvement in signal-to-noise ratio. Here, an alternative approach is proposed where the source wavelength is modulated (e.g., by a few nanometers) at a fixed frequency. Variation in the tissue absorption over the wavelength range will result in a small modulation in detected intensity, which can also be measured using lock-in or Fourier methods. As will be shown below, a potential advantage of this approach is a facility to estimate absolute concentrations of chromophores if unknown surface coupling losses and geometrical factors are insensitive to small changes in wavelength.

Wavelength modulated spectroscopy (WMS) has been widely used for many years to characterize molecular compositions of gases, particularly in hostile environments.^3^^,^^4^ WMS uses derivatives to emphasize subtle spectral features, an idea that has been attributed to Ernest Rutherford in the 1920s.^5^ Britton Chance is believed to have been the first to use the wavelength modulation principle in spectrophotometry in the 1940s,^3^ and the first application of spectral derivatives to *in vivo* NIRS measurements was by Ferrari et al.^6^ in 1989. More recently, Dehghani et al.^7^^,^^8^ have noted the insensitivity of spectral derivatives to unknown coupling coefficients for both spectroscopy and imaging, and have investigated the use of spectral derivatives for quantitative bioluminescence tomography.^9^

The potential of wavelength modulated NIRS is explored here by considering the modified Beer–Lambert law.^10^ This says that for a single source fiber and a single detector fiber placed on the surface of the interrogated tissue, separated by a distance d, the detected intensity is given as

$$I=kI_{0}e^{-\mu_{a}\beta d+G},$$ (1)

where $\mu_{a}$ is the absorption coefficient of the tissue, β is the differential pathlength factor (DPF), G is an unknown scattering-dependent geometric factor, $I_{0}$ is the intensity of the source, and k is the (usually unknown and highly variable) coupling efficiency of light. The value of $\mu_{a}$ depends on the concentrations $c_{n}$ of the constituent chromophores as follows:

$$\mu_{a}=\underset{n}{\sum }\varepsilon_{n}c_{n},$$ (2)

where n is the number of chromophores and $\varepsilon_{n}$ are their specific absorption coefficients. The specific absorption coefficient (defined to base e) is equal to the specific extinction coefficient (defined to base 10) multiplied by ln10. Because k and G are unknown, NIRS typically relies on measuring changes in intensity in response to changes in tissue optical properties under conditions where it is assumed that k and G remain constant. Thus a change in the concentrations of chromophores can be obtained from a change in the log intensity as follows:

$$\Delta \ln\;I=-\beta d.\Delta \mu_{a}=-\beta d.\underset{n}{\sum }\varepsilon_{n}\Delta c_{n}.$$ (3)

A combination of measurements at a minimum of n wavelengths enables the changes in concentration to be evaluated assuming we have a realistic estimate of β.

By differentiating the modified Beer–Lambert law equation with respect to wavelength, the following expression is obtained that represents the relative change in intensity per unit change in wavelength:

$$(\frac{1}{I})\frac{\partial I}{\partial \lambda }=(\frac{1}{k})\frac{\partial k}{\partial \lambda }+(\frac{1}{I_{0}})\frac{\partial I_{0}}{\partial \lambda }+\frac{\partial G}{\partial \lambda }-\beta d\frac{\partial \mu_{a}}{\partial \lambda }-\mu_{a}d\frac{\partial \beta }{\partial \lambda }.$$ (4)

This is equivalent to the derivative of the natural logarithm of intensity with respect to wavelength:

$$(\frac{1}{I})\frac{\partial I}{\partial \lambda }\equiv \frac{\partial \;\ln\;I}{\partial \lambda }.$$ (5)

If it is assumed that the coupling term k and geometric factor G have weak dependence on wavelength, and therefore, the terms involving ∂k/∂λ and ∂G/∂λ are negligible compared to other terms in Eq. (4), then the equation can be approximated as

$$(\frac{1}{I})\frac{\partial I}{\partial \lambda }=(\frac{1}{I_{0}})\frac{\partial I_{0}}{\partial \lambda }-\beta d\frac{\partial \mu_{a}}{\partial \lambda }-\mu_{a}d\frac{\partial \beta }{\partial \lambda }.$$ (6)

Dehghani et al.^7^ have demonstrated experimentally that coupling is insensitive to small changes in wavelength, and the tissue scattering on which G depends is also relatively weakly wavelength-dependent over the NIR region compared to tissue absorption.^11^

The wavelength dependence of the detector must also be considered. This can be introduced by assuming that the measured intensity I(λ) is multiplied by a factor proportional to the detector responsivity r(λ), defined as the generated photocurrent per incident light power (mA per mW). This introduces an additional term into Eq. (6) as follows:

$$(\frac{1}{I})\frac{\partial I}{\partial \lambda }=(\frac{1}{I_{0}})\frac{\partial I_{0}}{\partial \lambda }+(\frac{1}{r})\frac{\partial r}{\partial \lambda }-\beta d\frac{\partial \mu_{a}}{\partial \lambda }-\mu_{a}d\frac{\partial \beta }{\partial \lambda }.$$ (7)

In practice, the combined wavelength dependences of both $I_{0}(\lambda )$ and r(λ) can be measured by coupling the source and detector together prior to a tissue measurement, such that the measured intensity $I_{cal}(\lambda )$ is proportional to their product $r(\lambda )I_{0}(\lambda )$. Differentiating this product yields

$$(\frac{1}{I_{cal}})\frac{\partial I_{cal}}{\partial \lambda }=(\frac{1}{I_{0}})\frac{\partial I_{0}}{\partial \lambda }+(\frac{1}{r})\frac{\partial r}{\partial \lambda }.$$ (8)

This calibration measurement can then be subtracted from Eq. (7) to produce a measurement that is corrected for the wavelength dependencies of both source and detector:

$$(\frac{1}{I}){\frac{\partial I}{\partial \lambda }}_{corr}=(\frac{1}{I})\frac{\partial I}{\partial \lambda }-(\frac{1}{I_{cal}})\frac{\partial I_{cal}}{\partial \lambda }.$$ (9)

Combining the above equations then yields

$$(\frac{1}{I}){\frac{\partial I}{\partial \lambda }}_{corr}=-d.\underset{n}{\sum }c_{n}(\beta \frac{\partial \varepsilon_{n}}{\partial \lambda }+\varepsilon_{n}\frac{\partial \beta }{\partial \lambda }).$$ (10)

The specific absorption coefficients $\varepsilon_{n}$ and their derivatives are already known or can be obtained with arbitrary precision from laboratory measurements on samples of chromophores. Thus, if β and its derivative ∂β/∂λ can be reliably estimated, the above equation might enable the absolute concentrations $c_{n}$ to be evaluated from measurements of ${\left(\partial I/\partial \lambda /I\right)}_{\mathrm{corr}}$ at a minimum of n different wavelengths.

Continuous-wave (CW) NIRS already relies on estimates of DPF β, whose wavelength dependence has been widely investigated. For example, Scholkmann and Wolf^12^ propose the following formula for the DPF of the frontal human head, for wavelengths in the range 690 to 832 nm:

$$\beta (\lambda ,A)=a_{1}+a_{2}A^{0.8493}+a_{3}\lambda^{3}+a_{4}\lambda^{2}+a_{5}\lambda ,$$ (11)

where A is the subject age (years), λ is wavelength (nm), $a_{1}=223.3$, $a_{2}=0.05624$, $a_{3}=-5.723\times 10^{-7}$, $a_{4}=0.001245$, and $a_{5}=-0.9025$. The model was developed using least-squares fitting to data published by University College London (UCL) in 1996 and is shown to be consistent with other published data. Differentiating Eq. (11) yields

$$\frac{\partial \beta }{\partial \lambda }(\lambda )=3a_{3}\lambda^{2}+2a_{4}\lambda +a_{5}.$$ (12)

The values predicted by Eqs. (11) and (12) are plotted in Fig. 1, assuming a subject age of 25 years.

**Fig. 1:**
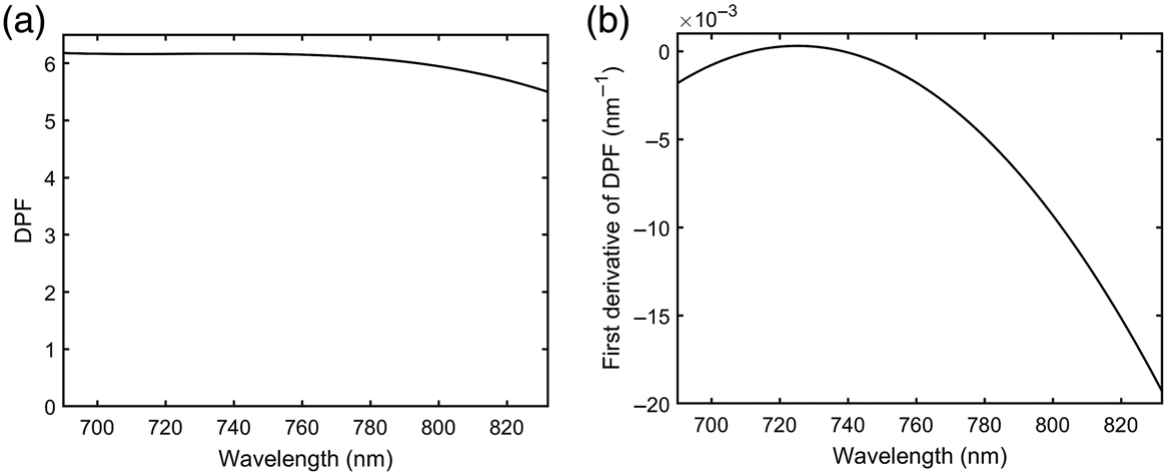
(a) Wavelength dependence of DPF and its (b) first derivative as predicted by the model of Scholkmann and Wolf for a subject age of 25 years.

A wavelength dependence of β is not surprising given that β depends on the absorption and the scattering of the tissue. It should be noted that the diffusion equation applied to a homogenous semi-infinite medium predicts that the DPF becomes virtually independent of d when $d\sqrt{3\mu_{a}\mu_{s}^{\prime }}\gg 1$, which typically holds when d>25  mm.^12^

Before considering how ∂I/∂λ/I can be measured in practice, a rough estimate is made of its likely magnitude for a measurement on the human brain. Suppose the absorption coefficient of the tissue is given as

$$\mu_{a,brain}=\epsilon_{HbO_{2}}c_{HbO_{2}}+\epsilon_{Hb}c_{Hb}+\mu_{a,H_{2}0}W+\mu_{a,Lipid}L+B,$$ (13)

where $\varepsilon_{HbO2}$ and $c_{HbO2}$ are the specific absorption coefficient and molar concentration of oxyhemoglobin, $\varepsilon_{Hb}$ and $c_{Hb}$ are the equivalent parameters for deoxyhemoglobin, $\mu_{a,H2O}$ and $\mu_{a,Lipid}$ are the absorption coefficients of water and lipid, W and L are the fractions of water and lipid in the brain, and B is a wavelength-independent background term. Estimates of the four coefficients as a function of wavelength are shown in Fig. 2 using data extracted from the online database compiled by Professor Scott Prahl of the Oregon Institute of Technology.^13^

**Fig. 2:**
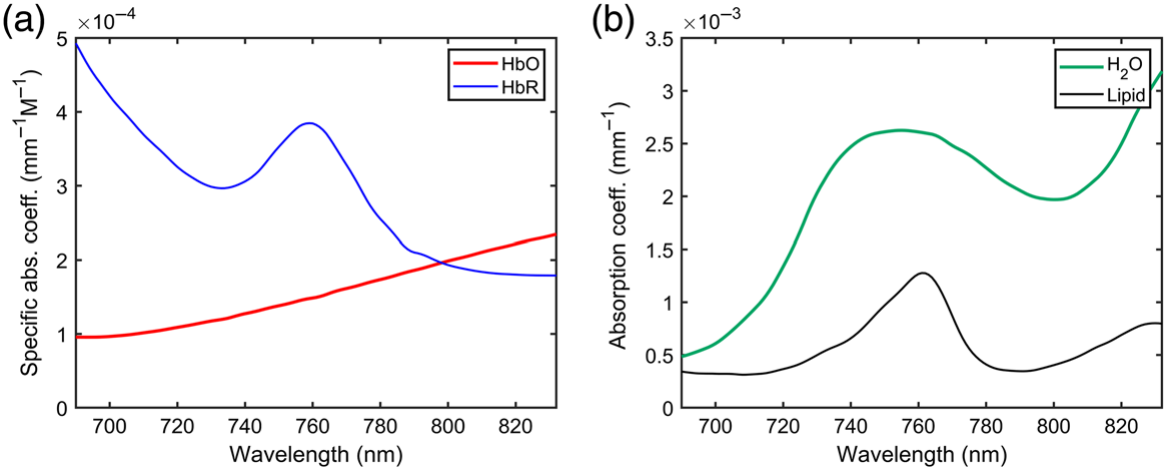
(a) Specific absorption coefficients of oxy- and deoxy-hemoglobin and (b) absorption coefficients of water and lipid.

Inserting Eq. (13) into Eq. (10) yields

$$(\frac{1}{I}){\frac{\partial I}{\partial \lambda }}_{\mathrm{corr}}=-d[(\beta \frac{\partial \varepsilon_{HbO_{2}}}{\partial \lambda }+\varepsilon_{HbO_{2}}\frac{\partial \beta }{\partial \lambda })c_{HbO_{2}}+(\beta \frac{\partial \varepsilon_{Hb}}{\partial \lambda }+\varepsilon_{Hb}\frac{\partial \beta }{\partial \lambda })c_{Hb}+(\beta \frac{\partial \mu_{a,H_{2}O}}{\partial \lambda }+\mu_{a,H_{2}O}\frac{\partial \beta }{\partial \lambda })W+(\beta \frac{\partial \mu_{a,\text{Lipid}}}{\partial \lambda }+\mu_{a,\text{Lipid}}\frac{\partial \beta }{\partial \lambda })L+B\frac{\partial \beta }{\partial \lambda }].$$ (14)

The nine terms inside the square brackets in Eq. (14) are sketched in Fig. 3(a) assuming reasonable estimates of $c_{HbO}=56\;\mu \mathrm{mol}$, $c_{Hb}=24\;\mu \mathrm{mol}$, W=0.8, L=0.116, $B=0.012\;{\mathrm{mm}}^{-1}$, and using Eqs. (11) and (12) to represent the wavelength dependence of β and ∂β/∂λ.

**Fig. 3:**
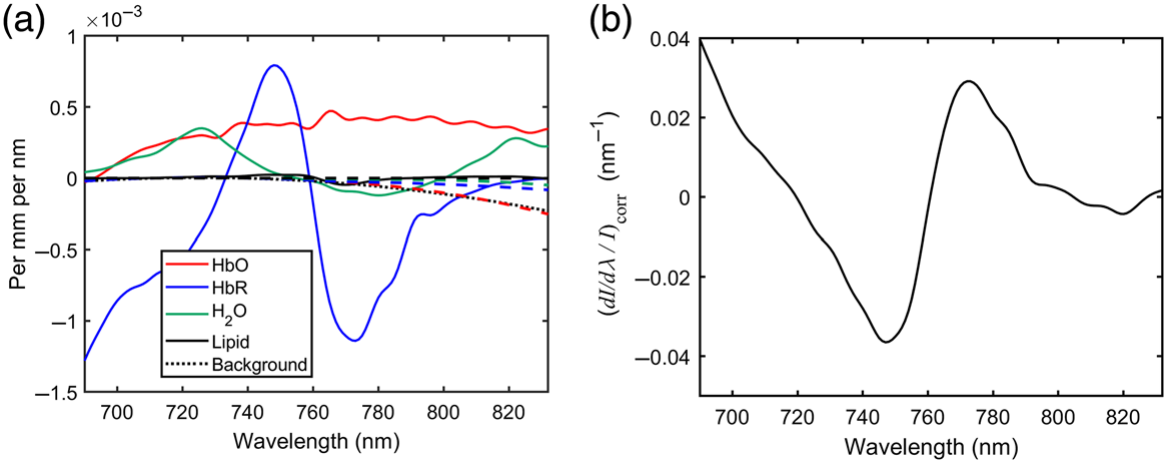
(a) Predicted contribution of the nine terms in Eq. (14) to a measurement of relative intensity modulation per nm change in wavelength of the brain; (b) Predicted relative intensity modulation per nm change in wavelength for a measurement on the human brain.

The solid lines represent the four terms containing β, and the dashed lines represent the five terms containing ∂β/∂λ. Figure 3(b) shows the relative change in intensity per unit change in wavelength ${\left(\partial I/\partial \lambda /I\right)}_{\mathrm{corr}}$ as a function of wavelength using the same data values and a source-detector separation d=30  mm.

This indicates a relative modulation that varies approximately between ±4% per nm over the displayed range of wavelengths. The overall profile exhibited in Fig. 3(b) is evidently dominated by the deoxyhemoglobin term. An experimental determination of the five unknown parameters in Eq. (14) ($c_{HbO2}$, $c_{Hb}$, W, L, and B) would require measurements at a minimum of five different wavelengths. Those wavelengths should be selected to enable the derivatives of the specific absorption coefficients to be easily distinguished from each other, and the optimum combination of wavelengths could be established using appropriate models.

Implementation of wavelength modulation NIRS requires a system that is able to measure two quantities: a small change in intensity resulting from a small perturbation in wavelength and the intensity at the central wavelength (or mean intensity over the narrow range of wavelengths). These quantities would need to be measured at several different wavelengths. To achieve the sensitivity enabled by lock-in or Fourier methods, the ideal source would therefore be both intensity and wavelength modulated (at different frequencies).

Four candidate technologies that could be used to evaluate the potential of the method are outlined below.

i Wavelength sweeping at kHz rates over several nanometers can be achieved using an acousto-optic tunable filter (AOTF) in combination with a broadband source, such as a supercontinuum (SC) laser. Simultaneous intensity modulation can be supplied using an external intensity modulator. The technology would be very flexible, allowing tuning over a broad range of NIR wavelengths, but is expensive.
ii The laser wavelength emitted by a vertical-cavity surface-emitting laser (VCSEL) can be rapidly scanned by varying the injection current. Again, an external intensity modulator would also be required. This is less expensive than the AOTF option but would be restricted to NIR wavelengths at which VCSELs were available.
iii Tunable diode laser (TDLs) are already widely used for WMS applications and can be tuned by either adjusting their temperature or by changing the injection current density. While temperature changes allow tuning over large bandwidths, it is limited by slow tuning rates (a few hertz), due to thermal inertia. Adjusting the injection current can provide tuning at GHz rates, but it is restricted to much smaller bandwidths. Quoted typical tuning coefficients are approximately 0.06  nm/°C by temperature or 0.003  nm/mA by current, and thus very accurate control of temperature or current is required, which will be technically challenging.

An experimental configuration for option (i) is illustrated schematically in Fig. 4(a). Options (ii) and (iii) can be configured by replacing the SC laser/AOTF with VCSEL or TDL sources, respectively.

iv As an alternative to wavelength sweeping, two or more discrete sources could be used, emitting at adjacent wavelengths (i.e., a few nm apart or less), fed into a single optical fiber. For two sources, Eq. (6) can be expressed as
  $$\Delta \;\ln\;I=\Delta \;\ln\;I_{0}-d.\Delta \lambda \underset{n}{\sum }c_{n}(\beta \frac{\partial \varepsilon_{n}}{\partial \lambda }+\varepsilon_{n}\frac{\partial \beta }{\partial \lambda }),$$ (15)
  where Δλ is the small difference between the wavelengths. One possible implementation would be to modulate the intensity of both sources at the same frequency while switching between them at a different frequency. Alternatively, instead of switching, the sources could be intensity modulated at different frequencies, and the wavelength-dependent difference in measured intensity is calculated by simply subtracting one from another. Laser diodes are widely available for a handful of discrete near-infrared wavelengths and have a spectral width of around 1 nm. The center wavelength for a given diode type can vary from production run to production run, and this variation may be sufficient to provide the laser diode pairs required. Alternatively, laser diodes can be temperature tuned to adjust the lasing wavelength^14^ (a measurement would be acutely sensitive to drift in laser wavelength). It is worthy to note that near-infrared light-emitting diodes have a typical width of a few tens of nm and therefore are probably not an option. A schematic for option (iv), involving two switched laser diode sources, is shown in Fig. 4(b).

**Fig. 4:**
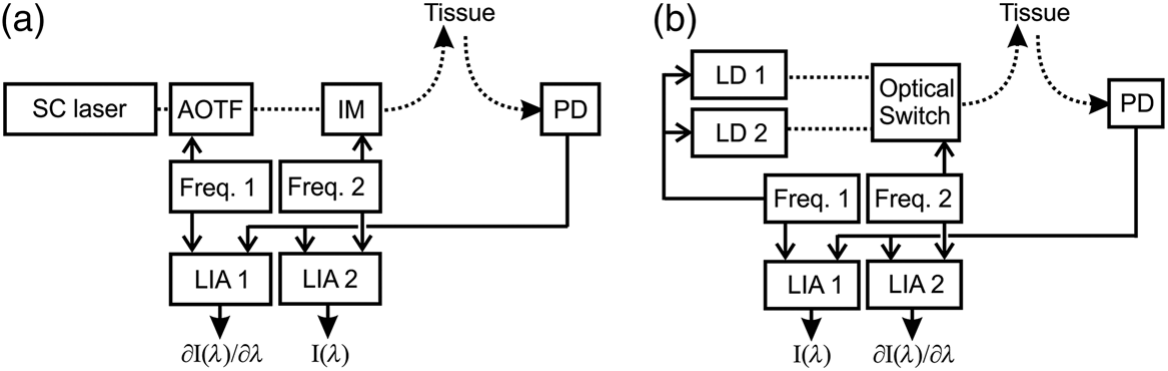
Experimental systems for evaluating the potential of wavelength modulated NIRS using (a) a SC laser and AOTF and (b) two laser diodes. The dashed line represents the light path, either via optical fibers or free space. [SC, supercontinuum; AOTF, acousto-optic tunable filter; IM, intensity modulator; PD, photodiode detector; LIA, lock-in amplifier; Freq. 1 and Freq. 2 generate reference signals at different frequencies, and drive the (a) AOTF and IM, and the LDs and the optical switch].

If wavelength modulated NIRS is to be quantitative, it will be essential to fully characterize the relationship between the measured quantities and the term ${\left(\partial I/\partial \lambda /I\right)}_{\mathrm{corr}}$ in Eq. (10). For example, lock-in amplifiers detect only the root-mean-square signal at the reference frequency, and the output depends on the shape of the oscillation waveform. For a sinusoidal modulation, this would introduce a factor of √2 between the output and the peak amplitude of the signal, but the factor would be different for non-sinusoidal modulation. This factor would need to be established for a given source.

When solving Eq. (10), not only is the measurement of relative intensity modulation ${\left(\partial I/\partial \lambda /I\right)}_{\mathrm{corr}}$ dependent on the spectral resolution of the system but so are the estimates of the chromophore specific absorption coefficients and their derivatives. If the “instrumental line profile” P(λ) is broad, then spectral features are smoothed out through convolution:

$$\epsilon_{meas}(\lambda )=\epsilon_{true}(\lambda )*P(\lambda ),$$ (16)

and therefore

$$\frac{\partial \epsilon_{meas}}{\partial \lambda }=\frac{\partial }{\partial \lambda }[\varepsilon_{true}*P]=\frac{\partial \epsilon_{true}}{\partial \lambda }*P(\lambda ).$$ (17)

Although the coefficients for intrinsic chromophores do not exhibit any narrow features within the near-infrared window, solving Eq. (10) is likely to involve error if the prior estimates and the measurements were recorded with significantly different spectral resolution.

The diagnostic potential of wavelength modulated NIRS depends on an easily-tested hypothesis that the relative modulation in detected intensity in response to a wavelength-modulated source is largely uninfluenced by changes in surface coupling. This, in itself, would appear to offer a significant advantage over established CW NIRS methods. However, a greater potential benefit of wavelength modulation would be a facility to estimate absolute concentrations of tissue chromophores. The analysis above suggests that this is possible, although technically challenging because of the dependency on many factors that require very careful calibration, such as the wavelength dependence of the source intensity and detector responsivity, and the spectral resolution. Estimates of absolute concentrations also depend on estimates of the DPF and its wavelength dependence. The Scholkmann and Wolf model reported above was based on data for the frontal human head, and will not apply to other anatomy due to known dependence on geometry.^15^ CW NIRS already relies on independent estimates (e.g., published values) of β, and wavelength modulated NIRS will additionally rely on estimates of ∂β/∂λ, although Fig. 3(a) suggests that the terms containing ∂β/∂λ may have a much smaller influence on intensity modulation than terms containing β over the displayed range of wavelengths. The dependence of DPF on wavelength, geometry, and the tissue optical properties may require independent measures of β and ∂β/∂λ using time-domain or frequency-domain technology in order to achieve sufficient quantitative accuracy for a given application.
